# Production of activated carbon from date palm stones by hydrothermal carbonization and microwave assisted KOH/NaOH mixture activation for dye adsorption

**DOI:** 10.1038/s41598-023-45864-z

**Published:** 2023-11-04

**Authors:** Saud S. Aloud, Hattan A. Alharbi, Bassim H. Hameed, John P. Giesy, Saad S. Almady, Khaled D. Alotaibi

**Affiliations:** 1 Department of Soil Science, College of Food and Agriculture Sciences, King Saud University, P.O. Box 2460, 11451 Riyadh, Saudi Arabia; 2 Department of Plant Protection, College of Food and Agriculture Sciences, King Saud University, P.O. Box 2460, 11451 Riyadh, Saudi Arabia; 3 Department of Chemical Engineering, College of Engineering, Qatar University, P.O. Box 2713, Doha, Qatar; 4 Department of Biomedical Sciences and Toxicology Centre, University of Saskatchewan, Saskatoon, SK S7N 5B3 Canada; 5 Department of Integrative Biology, Michigan State University, East Lansing, MI 48824 USA; 6 Department of Environmental Sciences, Baylor University, Waco, TX 76798 USA; 7 Agricultural Engineering Department, College of Food and Agriculture Sciences, King Saud University, 11451 Riyadh, Saudi Arabia

**Keywords:** Environmental sciences, Chemical engineering

## Abstract

Date palm stones are regarded as possible alternatives to activated carbon (AC) precursors with high potential for various environmental applications. In this research study, date palm stones derived activated carbon (DPSAC) was used as adsorbent for removing toxic remazol brilliant blue R (RBBR). The synthesis of DPSAC involved a chemical treatment using KOH and NaOH (1:1). Characterization of DPSAC revealed that it exhibited a BET surface area of 715.30 m^2^/g, Langmuir surface area of 1061.93 m^2^/g, total pore volume of 0.39 cm^3^/g, and average pore diameter of 2.15 nm. Adsorption uptake of RBBR increased (from 24.54 to 248.54 mg/g), whereas the removal percentage decreased (from 98.16 to 82.85%) when the initial RBBR concentration increased (from 25 to 300 mg/L). The adsorption process performed best under acidic conditions (pH 3), with an RBBR uptake of 98.33 mg/g. Because of the high R^2^ values (0.9906 and 0.9779) and low average errors (6.24 and 13.95%), this adsorption process followed the Freundlich isotherm and pseudo-first-order (PFO) models, respectively. The Langmuir adsorption capacity (Q_m_) was 319.63 mg/g. Thermodynamic parameters were − 11.34 kJ/mol for ∆H° (exothermic in nature), 0.05 kJ/mol K for ∆S° (increasing randomness level at solid–liquid interface), − 27.37 kJ/mol for ∆G° (spontaneous), and 6.84 kJ/mol for E_a_ (controlled by physisorption).

## Introduction

Unlike natural dyes, which are often acquired from plants, synthetic dyes are developed using organic materials as the raw materials^1^. Because of their ability to provide a wide range of attractive colors, synthetic dyes are used in numerous industries, including textiles, paper, magazines, plastics, cosmetics, and food^2^. While the production of synthetic dyes is beneficial for these industries, the discharge of dye-containing wastewater into the environment can cause adverse effects on humans, wildlife, and the function and structure of aquatic ecosystems in general^3^. Because of their complex chemical structures, synthetic dyes are recalcitrant, resistant to biodegradation, and stable against light and heat. Cationic dyes possess cationic groups, while reactive cationic dyes contain extra reactive groups capable of interacting with fiber reactive groups, resulting in the formation of covalent bonds between the dye and fiber^4^.One of the most popular dyes used in the textile industry is remazol brilliant blue R (RBBR), a reactive dye that is chemically stable and has low energy consumption^5^. The presence of dyes in water can prevent sunlight from reaching the aquatic plants, thereby impeding photosynthesis^6^. Most importantly, RBBR is known to be toxic and carcinogenic to humans^7^.

This dye dissolves in water and generates negative ions that are attracted to the partially positive side of the polar molecules of water, which makes it difficult to remove it from water during treatment^8^; owing to the harmful effects caused by RBBR, various technologies, such as membrane filtration, chemical precipitation, coagulation-flocculation and adsorption, have been developed and employed to treat wastewater containing dyes^9^. Adsorption using activated carbon (AC) is among the most versatile technologies as it can removes a variety of contaminants, including synthetic dyes^10–13^, heavy metals^14,15^, phenolic compounds^16^, pesticides^17^ and other organic compounds. Moreover, this method is relatively inexpensive, and AC can be derived from various biomass wastes, including water hyacinth^18^, algae^19^, coconut shell^20^, apple waste^21^, andiroba shell^22^, durian peel^23^, tea residue^24^, pine sawdust^25^, and herbaceous plants^26^, among others. Some studies have shown that biomass-based AC are a feasible source of AC^10^, further reducing waste generated by factories and farming activities^27^. Finally, adsorption is a relatively fast process, with one study reporting that the equilibrium state can be attained in as fast as 45 min^28^.

In this study, date palm stone was used to produce AC (DPSAC) through chemical activation using a mixture of KOH and NaOH under microwave heating. The date palm, *Phoenix dactylifera L* is a tree of the *Arecaceae* family. An estimated 40 kg of biomass waste is generated annually from this tree^29^. Date stones alone contribute to approximately 10–15% of the total mass of date fruits^30^. Recent studies have focused on activating samples using microwave irradiation as opposed to conventional heating in a furnace. Activation by microwave irradiation is rapid; therefore, this approach reduces the time and resources required for production. AC with attractive properties has been produced through chemical activation using a mixture of KOH and NaOH to remove heavy metals^31^ and basic dyes^30^. However, there have been no studies on the efficiency of the asdsorptoin of reactive dyes. Therefore, this study focuses on utilizing DPSAC to remove the reactive dye from aqueous solution.

## Results and discussion

### Characteristics of samples

Characteristics of DPSAC in terms of BET surface area and Langmuir surface area were determined to be 715.30 m^2^/g and 1061.93 m^2^/g, respectively. This BET surface area is relatively similar to that of common bamboo (*Bambusa vulgaris striata*)-based AC (BVSAC), which has a BET surface area of 1108 m^2^/g^32^. This is because BVSAC was chemically activated with KOH and NaOH at a greater impregnation ratio of 2:3:3 than DPSAC, which had an impregnation ratio of 2:1:1 for char:KOH:NaOH. Higher amounts of KOH and NaOH enhanced pore formation, thus increasing the surface area. In another study, where char was produced from date-stone-based AC (DSAC), a larger surface area of 1123 m^2^/g was observed because of the greater activation power used (850 W) compared with DPSAC (700 W). The use of greater radiation power increases the intensity of the degradation of polar compounds, such as lignin and hemicellulose, in biomass waste. The total power volume and average pore diameter of DPSAC were 0.39 cm^3^/g and 2.15 nm, respectively. As the pore size was between 2 and 50 nm, the pores in DPSAC were defined as mesopores.

Date palm stones contained high percentages of carbon (C) (41.77%) and fixed carbon (26.77%) (Table 1). The percentage of C in other biomass wastes, including seeds of Phoenix flower (*Delonix regia*)^33^ *acai* seeds, was 43.3%^34^. Microwave activation was effective in pyrolyzing the precursor, thereby reducing the percentage of moisture (from 5.75 to 3.38%) and volatile matter (from 66.54 to 12.45%). During microwave activation, the polar compounds in the precursor absorb microwave energy and vibrate at extremely high speeds. This vibration dissipates heat, which then reduces the moisture and volatile matter components. Consequently, the percentage of fixed carbon increased from 26.77 to 82.45%. The ash content was less than 2% for both the precursors and DPSAC. This ash percentage is advantageous for the adsorbent because ash contains no pores and does not participate in adsorption.

**Table 1:** Elemental analysis of samples.

Samples	Elemental analysis (%)					Proximate analysis (%)			
	C	H	N	S	Others	Moisture	Volatile matter	Fixed carbon	Ash
Precursor	41.77	7.42	1.67	0.31	48.83	5.75	66.54	26.77	0.94
DPSAC	74.18	2.03	0.63	0.04	23.12	3.38	12.45	82.45	1.71

**Figure 1:**
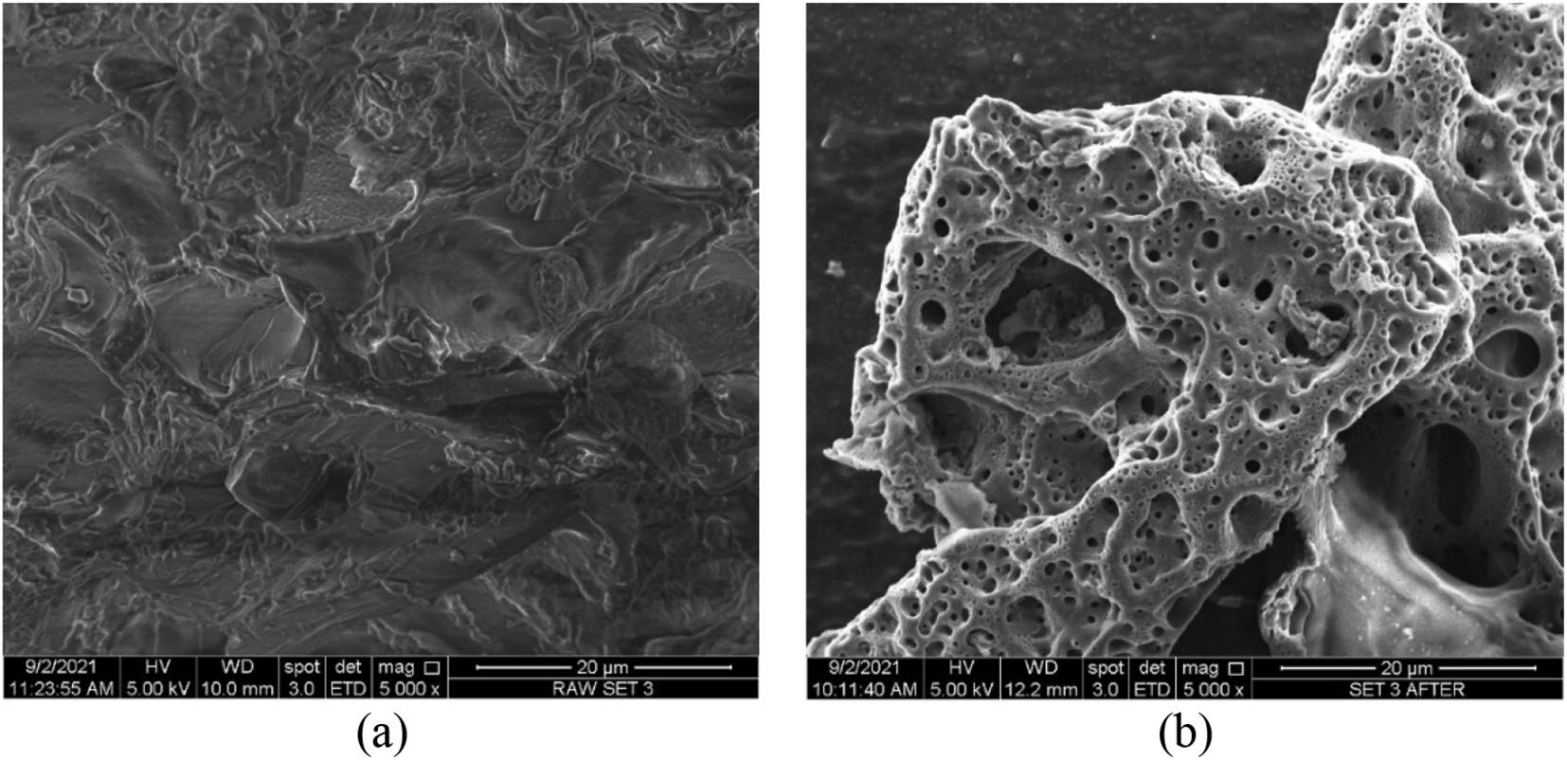
SEM images for (**a**) precursors and (**b**) DPSAC (5000 × magnification).

The following characterization of the samples was performed in terms of surface morphology (Fig. 1a,b) and shows the SEM images of the precursor and DPSAC at a magnification level of 5000x. The surface morphology of the precursor was dense with no pores. As against, the surface morphology of DPSAC was filled with numerous randomly distributed pores. These pores were dissimilar in size, which was attributed to the use of two chemicals, KOH and NaOH, during the chemical activation step. The AC derived from *B. vulgaris striata* also displayed various pore sizes (micropores and mesopores) after undergoing KOH/NaOH chemical activation^32^. Several possible chemical reactions occur during chemical activation^39,40^ (Eqs. 1–13).

1 $${\text{2KOH }}\left( {{\text{aq}}} \right) \to {\text{K}}_{{2}} {\text{O}}\left( {\text{s}} \right) + {\text{H}}_{{2}} {\text{O}}\left( {\text{l}} \right)$$

2 $${\text{2NaOH}}\left( {{\text{aq}}} \right) \to {\text{Na}}_{{2}} {\text{O}}\left( {\text{s}} \right) + {\text{H}}_{{2}} {\text{O}}\left( {\text{l}} \right)$$

3 $${\text{H}}_{{2}} {\text{O}}\left( {\text{l}} \right) + {\text{C}}\left( {\text{s}} \right) \to {\text{CO}}\left( {\text{g}} \right) + {\text{H}}_{{2}} \left( {\text{g}} \right)$$

4 $${\text{CO}}\left( {\text{g}} \right) + {\text{H}}_{{2}} {\text{O}}\left( {\text{l}} \right) \to {\text{CO}}_{{2}} \left( {\text{g}} \right) + {\text{H}}_{{2}} \left( {\text{g}} \right)$$

5 $${\text{CO}}_{{2}} \left( {\text{g}} \right) + {\text{K}}_{{2}} {\text{O}}\left( {\text{s}} \right) \to {\text{K}}_{{2}} {\text{CO}}_{{3}} \left( {\text{s}} \right)$$

6 $${\text{CO}}_{{2}} \left( {\text{g}} \right) + {\text{Na}}_{{2}} {\text{O}}\left( {\text{s}} \right) \to {\text{Na}}_{{2}} {\text{CO}}_{{3}} \left( {\text{s}} \right)$$

7 $${\text{K}}_{{2}} {\text{CO}}_{{3}} \left( {\text{s}} \right) \to {\text{K}}_{{2}} {\text{O }}\left( {\text{s}} \right) + {\text{CO}}_{{2}} \left( {\text{g}} \right)$$

8 $${\text{Na}}_{{2}} {\text{CO}}_{{3}} \left( {\text{s}} \right) \to {\text{Na}}_{{2}} {\text{O }}\left( {\text{s}} \right) + {\text{CO}}_{{2}} \left( {\text{g}} \right)$$

9 $${\text{CO}}_{{2}} \left( {\text{g}} \right) + {\text{C}}\left( {\text{s}} \right) \to {\text{2CO}}\left( {\text{g}} \right)$$

10 $${\text{K}}_{{2}} {\text{O}}\left( {\text{s}} \right) + 2{\text{C}}\left( {\text{s}} \right) \to {\text{K}}\left( {\text{s}} \right) + {\text{2CO}}\left( {\text{g}} \right)$$

11 $${\text{Na}}_{{2}} {\text{O}}\left( {\text{s}} \right) + {\text{2C}}\left( {\text{s}} \right) \to {\text{Na}}\left( {\text{s}} \right) + {\text{2CO}}\left( {\text{g}} \right)$$

12 $${\text{K}}_{{2}} {\text{CO}}_{{3}} \left( {\text{s}} \right) + {\text{C}}\left( {\text{s}} \right) \to {\text{2K}}\left( {\text{s}} \right) + {\text{3CO}}\left( {\text{g}} \right)$$

13 $${\text{Na}}_{{2}} {\text{CO}}_{{3}} \left( {\text{s}} \right) + {\text{C}}\left( {\text{s}} \right) \to {\text{2Na}}\left( {\text{s}} \right) + {\text{3CO}}\left( {\text{g}} \right)$$

Three main steps occur during the chemical activation process using KOH/NaOH^35,36^. The first step involves the formation of CO, CO_2_, and H_2_O (Eqs. 12–15), where pore initiation occurs via the gasification of the carbon matrix. The next step is the production of salts, namely K_2_O, Na_2_O, K_2_CO_3_, and Na_2_CO_3_ (Eqs. 16–19). These salts form at temperatures above 700 °C where the pores develop through redox reaction. Finally, pores are formed through the diffusion and integration of metallic K^+^ and Na^+^ (Eqs. 21–24) in the graphitic layers of the samples.

**Figure 2:**
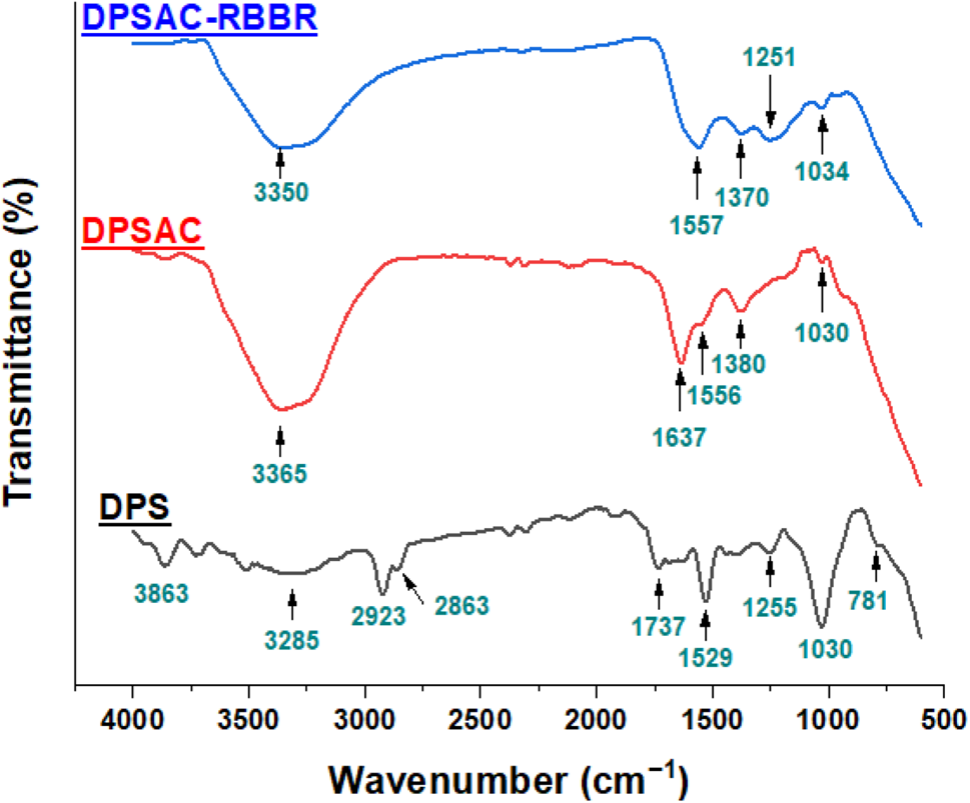
FTIR spectra for date palm stones (DPS) precursor, DPSAC-before and DPSAC-after adsorption of remazol brilliant blue R (RBBR).

The Fourier Transform Infrared (FTIR) spectroscopy spectra of the precursor (DPS), DPSAC, and RBBR-loaded DPSAC adsorbent (DPSAC-RBBR) have been presented in Fig. 2, and their characteristic structural bands in Table 2. As the results suggest, the spectral profile of DPS is the most crowded over the analyzed range of 650–4000 cm^−1^. This is typically in agreement with the literature^37–39^. It shows a broad peak centered at 3285 cm^−1^ for the OH group of glucose (alcohols), phenols, and water molecules; a weak band around 3100 cm^−1^ traces the =C–H aromatic compounds and strong vibrations at 2923 and 2863 cm^−1^ indicate the asymmetric and symmetric aliphatic C–H (carbon-*sp*^3^) modes of cellulose, hemicellulose, and lignin^40^. The peak at 1737 cm^−1^ refers to the carbonyl (C=O) group of esters. Furthermore, the vibrational bands at 1624 and 1529 cm^−1^ can be assigned, respectively, to C=N and C=C stretching’s. The prominent peaks at 1255 and 1030 cm^−1^ are attributed to the stretching vibration of C–O and C–O–C. The bending vibrations of alkyl C–H bonds are visible over the fingerprint region, for example, at 1391 and 781 cm^−1^. The overlapping of the peaks was due to the presence of multiple types of organic compounds, which are cellular OH-rich phytocompounds that may cause absorption saturation. After carbonization, new peaks were heightened, whereas the other peaks were diminished. Hence, the peak of OH in the DPSAC spectrum became broader (centered at 3365 cm^−1^) due to its absorptivity compared to other bonds, further supporting its abundance on the surface of the activated carbon. Hydrothermal treatment and the application of KOH/NaOH chemical activators have been reported to enrich the surface of the AC with oxygen-containing functional groups^38^. However, peaks for C–H alkyls, typically at 2800–2990 cm^−1^, were disappeared due to carbonization to activate carbon in which the aromatic structure is dominant. The disappearance of the band at 1737 cm^−1^ suggests the decomposition of hemicellulose. In addition, the strong band at 1030 cm^−1^, attributed to the C–O asymmetric stretching vibration in cellulosic compounds, was diminished. However, the retained low-intensity peak is related to the presence of the C–O functional group, which was further supported by the symmetric band at 957 cm^−1^^41^. Other bands corresponding to C=C, C=N, and C–H were seen at 1637, 1556, and 1380 cm^−1^, respectively. The peaks at 1637 and 1556 cm^−1^ overlapped and slightly shifted to 1557 cm^−1^, whereas the red peak at 1380 cm^−1^ shifted to 1370 cm^−1^ after adsorption. In addition, new, high intense band at 1250 cm^−1^ was observed. The new bands can be ascribed to the adsorbed RBBR dye, whereas the shift indicates the participation of C=C, C=N, OH, and C–O groups in the adsorption mechanism.

**Table 2:** Characteristic bands for DPS precursor, DPSAC-before and DPSAC-after adsorption of RBBR.

Peak assignment	DPS	DPSAC	DPSAC-RBBR
ν(O–H) alcohols, phenols	3285, br	3365, br	3350, br
ν(C–H) aromatic ring	3100 vw	–	–
ν(C–H) aliphatic: methyl and methylene, cutin, waxes	2923 asym, s; 2863 sym, s	–	–
ν(C=O) ester: acetyl and uronic ester of hemicelluloses,	1737 m,	–	–
ν(C=C), ν(C=N) aromatic rings	1624 w,	1637 s	–
ν(C=C) aromatic skeletal	1529 s,	1556 m	–
δ(C–H) cellulose	1391 m	1380 s	1370 m
ν(C–O) phenolic;	1255 m	1190 w	1251 s
ν(C–O) and ν(C–O–C) of cellulose and hemicellulose	1030 vs	1030 m	1034 m
ρ(C–H) cellulose	781 w	–	–

asym, asymmetric vibration; sym, symmetric vibration; br, broad; w, weak; m, medium; s, strong; vw, very weak. Band type: ν, stretching; ν, bending; ν, rocking.

### Adsorption equilibrium

**Figure 3:**
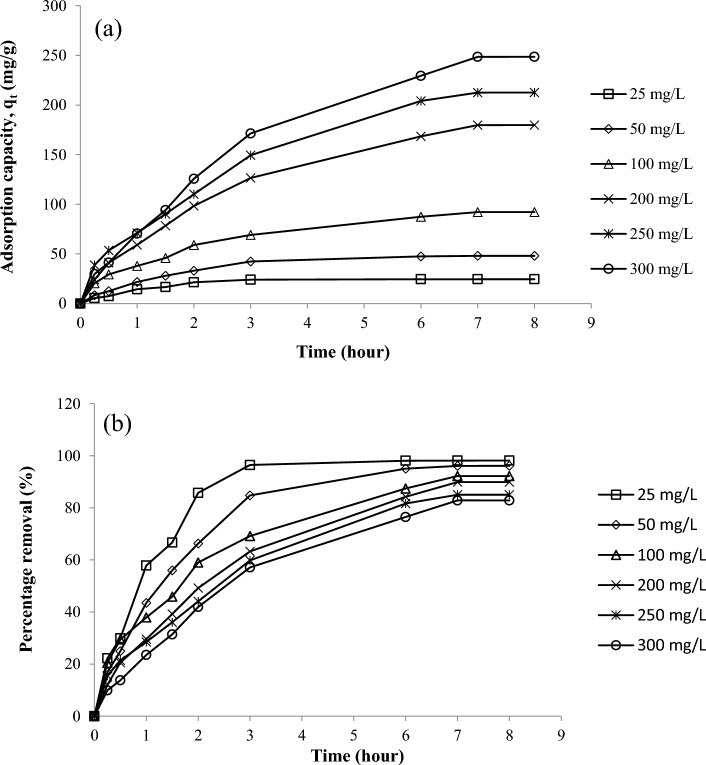
(**a**) Adsorption of RBBR by DPSAC versus time at 30 °C for different initial concentration (0.2 g adsorbent dosage and 200 ml of solution at its original pH); (**b**) Removal of RBBR by DPSAC versus time at 30 °C for different initial concentration (0.2 g adsorbent dosage and 200 ml of solution at its original pH).

The initial concentration and contact time of the adsorbents are the two most important factors affecting adsorption. Figure 3a shows the plots of RBBR uptake by DPSAC as a function of contact time for various initial concentrations of RBBR and Fig. 3b shows the plots of the percentage of RBBR removal. As the contact time increased, the uptake of RBBR increased in the early phases of adsorption (Fig. 3a), after which the uptake of RBBR became static, indicating that equilibrium had been reached. At that point, DPSAC was already exhausted, and no more RBBR was available for adsorption. At lower concentrations of RBBR, including 25 and 50 mg/L, 3 h were required to reach equilibrium, whereas at higher concentrations of RBBR, including 100, 200, 250, and 300 mg/L, 7 h were required. At lower concentrations, fewer RBBR molecules were available, such that adsorption reached equilibrium more quickly. Due to the fact that higher amount of RBBR molecules were available in the solution at greater initial concentrations of RBBR of 300 mg/L, adsorption of RBBR was 248.54 mg/g compared to 24.54 for 25 mg/L. In contrast, the percentage removal of PBBR at an initial concentration of 25 mg RBBR/L was 98.16%, but it was 82.85% at an initial concentration of 300 mg PBBR/L. At higher initial concentrations of PBBR, the ratio of RBBR molecules to accessible adsorption sites on DPSAC was large; thus, competition among the RBBR molecules for adsorption by DPSAC was greater. Consequently, a lower percentage of RBBR removal was observed at higher initial concentrations of PBBR.

**Figure 4:**
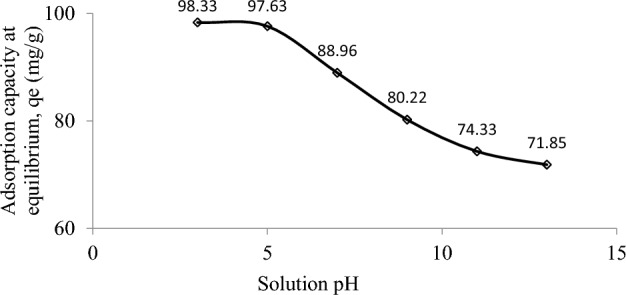
Adsorption of RBBR by DPSAC as a function of pH at 30 °C (100 mg/L initial concentration, 0.2 g adsorbent dosage and 200 ml of solution shaken for 8 h).

Another parameter that can affect adsorption is pH. The plots of RBBR adsorption over a range of pH values are presented in Fig. 4. Adsorption of 71.85 mg RBBR/g was observed at pH 13. The existence of many OH^−^ ions causes the surface of DPSAC to become negatively charged, thus repelling the anionic RBBR. At pH 11, 9, and 7, the adsorption capacities of RBBR were 74.33, 80.22 and 88.96 mg/g, respectively. At pH 5, the adsorption of RBBR was 97.63 mg/g since excess H^+^ in the solution resulted in the surface of DPSAC being positively charged, enhancing the attraction between the surfaces of the anionic DPSAC and RBBR. Further decreasing the pH of the solution to 3 only increased the adsorption of RBBR uptakes to 98.33 mg/g, indicating that the induction effect caused by H^+^ ions had reached its optimum value. At this optimum state, adding more H^+^ to the solution did not affect the adsorption of RBBR.

### Adsorption isotherm

**Figure 5:**
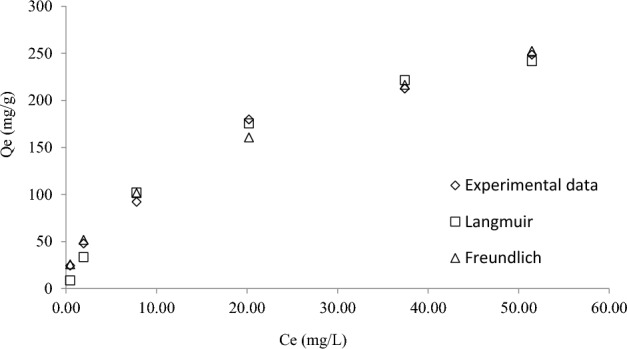
Plots of isotherm models for RBBR-DPSAC adsorption system at 30 °C.

The experimental data were described using Langmuir and Freundlich isotherms (Fig. 5 and Table 3). The adsorption of RBBR onto DPSAC was best fitted by the Freundlich model (correlation coefficient, R^2^ = 0.9906 and a lower root means square errors, RMSE = 9.09). This indicates that a multilayer coverage of RBBR molecules was formed on the surface of the DPSAC. The monolayer adsorption capacities obtained from the Langmuir model, Q_m_, was 319.63 mg/g. This value is large when compared to removal of RBBR by Jhingan gum hydrogel of 9.88 mg/g^42^ and sewage sludge biochar of 126.59 mg/g^43^. The heterogeneity factor n was 2.07, with a value being between 1 and 10 indicating a favorable adsorption process^44^.

**Table 3:** Isotherm parameters for RBBR-DPSAC adsorption system at 30 °C.

Isotherm	Parameters	30 °C
Langmuir	Q_m_	319.63
	K_L_	0.06
	R^2^	0.9879
	RMSE	10.87
Freundlich	K	37.65
	n	2.07
	R^2^	0.9906
	RMSE	9.09

### Adsorption kinetics

**Figure 6:**
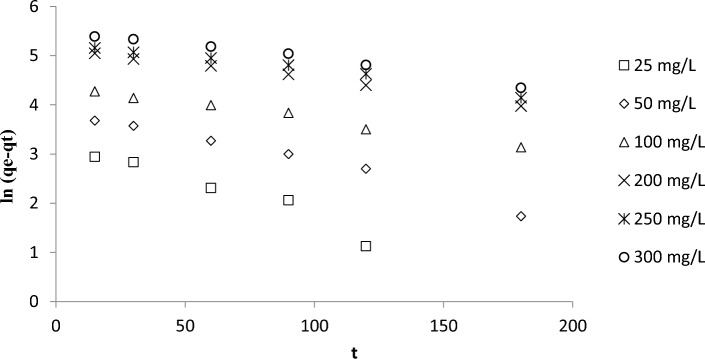
Plots of PFO kinetic model for RBBR-DPSAC adsorption system at 30 °C.

**Figure 7:**
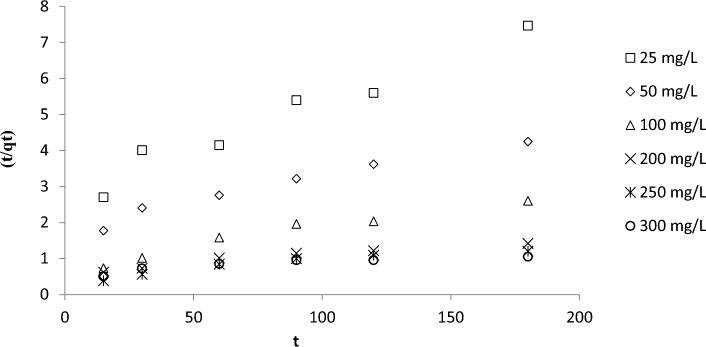
Plots of PSO kinetic model for RBBR-DPSAC adsorption system at 30 °C.

Kinetics were described by assuming the PFO (Fig. 6 and Table 4) and PSO models (Fig. 7 and Table 4). PFO provided the best kinetic fit for the adsorption of RBBR on DPSAC (RMSE = 10.11, R^2^ values between 0.9439 and 0.9949). Other dyes that were removed by AC derived from the leguminous tree *Adenanthera paronina*^45^ and the shells of the drumstick tree *Moringa Oleifera*^46^ were also best described by assuming a PFO. Increasing the initial concentration of RBBR from 25 to 300 mg/L decreased k_1_ and k_2_ from 0.0227 to 0.0063 min^−1^ and from 0.00025 to 0.00001 g mg^−1^ min^−1^, respectively. Higher initial concentrations of RBBR resulted in more RBBR molecules being present in the solution, which led to greater competition for adsorption sites on DPSAC. Consequently, the rate of adsorption was inversely related to the initial concentration of RBBR, which produced lesser values of k_1_ and k_2_.

**Table 4:** Kinetic parameters for RBBR-DPSAC adsorption system at 30 °C.

C_o_ (mg/L)	q_e_, exp (mg/g)	Pseudo-first order (PFO)				Pseudo-second order (PSO)			
		q_e_, cal (mg/g)	k_1_ (min^−1^)	RMSE	R^2^	q_e_, cal (mg/g)	k_2_ (g mg^−1^ min^−1^)	RMSE	R^2^
25	24.53	36.87	0.0227	10.11	0.9439	38.17	0.00025	42.57	0.9542
50	48.05	51.58	0.0115		0.9782	70.92	0.00011		0.9646
100	92.20	80.05	0.0069		0.9886	90.91	0.00016		0.9442
200	179.79	173.45	0.0064		0.9949	208.33	0.00004		0.9347
250	212.57	197.31	0.0060		0.9781	204.08	0.00005		0.8805
300	248.53	253.81	0.0063		0.9834	344.83	0.00001		0.7904

### Thermodynamics of adsorption

**Figure 8:**
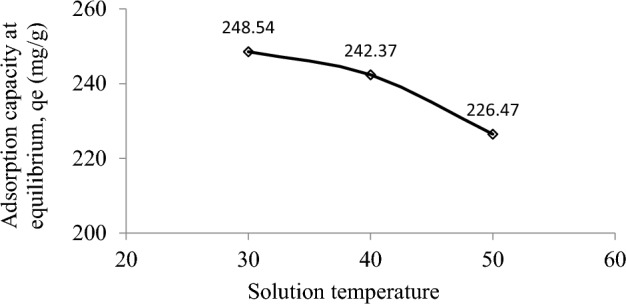
Adsorption of RBBR by DPSAC versus different solution temperature (300 mg/L initial concentration, 0.2 g adsorbent dosage and 200 ml of solution).

The verification of thermodynamic nature of RBBR-DPSAC adsorption process was verified by performing the adsorption process at three distinct temperatures: 30, 40, and 50 °C. The RBBR uptakes were found to decrease from 248.54 to 226.47 mg/g when the solution temperature was raised from 30 to 50 °C, thus signaling an exothermic nature (Fig. 8, Table 5). This result was reliable since the value for change in enthalpy (∆H°) was − 11.34 kJ/mol. The negative value of this parameter indicates that the adsorption process is exothermic. An exothermic nature was also observed in the adsorption of Reactive Red 141 dye by cotton-fiber-based AC^47^ and the adsorption of Reactive Red 195 onto surface-modified lychee peels^48^. The value of change in entropy (∆S°) was 0.05 kJ/mol. K, and the positive sign indicates an increase in the randomness at the liquid–solid interface. The value for Arrhenius activation energy (E_a_) was 6.84 kJ/mol and since this value was less than 40 kJ/mol, adsorption of RBBR onto DPSAC was controlled by physisorption^49^. Lastly, Gibbs energy (∆G°) values were determined to be − 27.37, − 27.90, and − 28.43 kJ/mol at 30, 40, and 50 °C, respectively. These negative signs verify that the adsorption process is naturally spontaneous at all solution temperatures studied.

**Table 5:** Thermodynamic parameters.

Temperature (°C)	∆H° (kJ/mol)	∆S° (kJ/mol K)	Ea (kJ/mol)	∆G° (kJ/mol)
30	− 11.34	0.05	6.84	− 27.37
40				− 27.90
50				− 28.43

## Materials and method

### Materials

The raw date stones used in the current study were a waste product of date palm fruits collected from local consumers in Riyadh, Saudi Arabia. All chemicals, namely sodium hydroxide (NaOH) and potassium hydroxide (KOH), were procured from Sigma Aldrich while 0.10 M HCl was provided by R&M Chemicals. The adsorbate of the RBBR dye in powder form was supplied by Merck. Inert gas of N_2_ was purchased from MOX Gases (Berhad).

### Preparation of activated carbon from date palm stones (DPSAC)

Raw date palm stones were cleaned with tap water, then dried in an oven for 24 h at 70 °C for 48 h. The dried materials were ground to a particle size of 1.0–2.0 mm. The precursor was converted into hydrochar by hydrothermal carbonization at 200 °C for 5 h. The resulting hydrochar was washed gently and dried in an oven. The hydrochar was then impregnated with NaOH and KOH in a ratio (IR) of 2:1:1 (w/v/v). The impregnation was performed for 8 h. Subsequently, the sample was dried again under the previous conditions. Next, the dried, impregnated hydrochar was activated in a microwave oven at 700 W for 10 min under a flow of N_2_ gas at 80 cm^3^/min. The following step required the activated sample to be soaked with 0.10 M HCl acid for 30 min under stirring. The sample was then washed with warm water until the pH of the washing solution reached 6–7. Next, the wet samples were oven-dried. Once dried, the sample was classified as date palm stone-based AC (DPSAC).

### Characterization methods

The DPSAC was characterized based on the Brunauer–Emmett–Teller (BET) surface area, Langmuir surface area and total pore volume in addition to average pore size using a volumetric adsorption analyzer (Micromeritics ASAP 2020). DPSAC was visually characterized using scanning electron microscopy (SEM) (LEO SUPRA 55VP, Germany), followed by elemental and proximate analyses using a simultaneous thermal analyzer (STA) (Model Perkin Elmer STA 6000, USA) and a thermogravimetric analyzer (TGA)^4,50^, respectively. The functional groups of DPSAC was analyzed using FTIR spectroscopy (IR Prestige 21 Shimadzu, Japan)^4^. The zeta potential distributions of the samples were obtained from a characterization test using a zeta potential analyzer (Zetasizer Nano Series DKSH).

### Equilibrium study

The adsorption equilibrium was investigated by changing the values of several parameters to determine their effects on adsorbent performance in removing the adsorbate. The effects of adsorbent concentration were studied by varying the initial concentration of RBBR. Six RBBR solutions with known initial concentrations of 25–300 mg/L were prepared by diluting the RBBR stock solution (1000 mg/L) with deionized water. These six solutions were prepared in six conical flasks and placed in a water bath shaker. Afterward, 0.2 g of DPSAC was added into each flask. The mouths of the conical flasks were sealed with a sealing film to prevent water loss by evaporation. Conditions of the water bath shaker were 30 °C and 60 °C. A small quantity of the sample was withdrawn from the conical flasks every 30 min, and the concentration of DPSAC was determined by UV–Vis spectrophotometry (Agilent Cary 60, USA). This process was allowed to proceed until adsorption reached equilibrium. Effect of temperature on adsorption was determined at three temperatures, 30, 40, and 50 °C without any pH adjustment. The effects of pH on adsorption were studied under both acidic and alkaline conditions over the pH ranges of 3, 5, 7, 9, 11, and 13. The solution pH was changed with the aid of HCl or NaOH while its temperature was maintained at 30 °C. Throughout the investigation of the effects of solution temperature and pH, other experimental conditions, such as the solution volume, DPSAC weight, and shaking speed of the water bath shaker, were fixed at 200 mL, 0.2 g, and 60 rpm, respectively. The DPSAC adsorption capacity for RBBR molecules and RBBR removal percentage were calculated using Eqs. (14 and 15).

14 $$q_{e} = \frac{{\left( {C_{o} - C_{e} } \right)V}}{M}$$

15 $$Removal \left( \% \right) = \frac{{\left( {C_{o} - C_{e} } \right)}}{{C_{o} }} \times 100\%$$

where the RBBR molecules removed by DPSAC at equilibrium stage (mg/g), the initial concentration of RBBR (mg/L), the equilibrium concentration of RBBR (mg/L), the volume of RBBR solution, and the weight of DPSAC used are denoted by q_e_, C_o_, C_e_, V, and M, respectively.

### Isotherm study

The relationship of the adsorbate concentration between the two phases (solid bulk) was verified by studying isotherm models. Therefore, the Langmuir and Freundlich models were selected for the purpose of this study (Eqs. 16 and 17).

Langmuir^51^:

16 $$q_{e} = \frac{{Q_{m} K_{L} C_{e} }}{{1 + K_{L} C_{e} }}$$

Freundlich^52^:

17 $$q_{e} = K_{F} C_{e}^{{{\raise0.7ex\hbox{$1$} \!\mathord{\left/ {\vphantom {1 {n_{F} }}}\right.\kern-0pt} \!\lower0.7ex\hbox{${n_{F} }$}}}}$$

where the Langmuir monolayer adsorption capacity (mg/g), Langmuir constant involving adsorption energy (L/mg), Freundlich constant of the adsorption process ((mg/g)(L/mg)^1/n^), Freundlich heterogeneity factor, universal gas constant (8.315 J/mol K), and the temperatures of the RBBR solution are denoted by Q_m_, K_L_, K_F_, n_F_, R, and T, respectively. The nonlinear equations of these isotherm models were solved using Microsoft Excel Solver v. 2016. The values of the R^2^ and the RMSE were determined to identify the best model according to the adsorption process. The RMSE value was calculated using^53^ Eq. (18).

18 $$RMSE = \sqrt {\frac{1}{n - 1}} \mathop \sum \limits_{n = 1}^{n} \left( {q_{e,exp,n} - q_{e,cal,n} } \right)^{2}$$

### Kinetic study

Parameters used for the kinetic study were similar to those of the isotherm study. However, in the kinetic study, the samples were withdrawn for concentration determination within a predetermined time range (0–180 min). Hence, the two most commonly employed kinetic models, namely the pseudo-first order (PFO)^54^ and pseudo-second order (PSA) models (Eqs. 19 and 20), were applied in this study.

19 $$q_{t} = q_{e} \left[ {1 - exp\left( { - k_{1} t} \right)} \right]$$

20 $$q_{t} = \frac{{k_{2} q_{e}^{2} t}}{{1 + k_{2} q_{e} t}}$$

where the PFO rate constant (min^−1^) and PSO rate constant (g mg^−1^ min^−1^) are denoted as k_1_ and k_2_, respectively. The best model for describing the kinetic data was selected based on R^2^ and RMSE values.

### Thermodynamic study

The adsorption efficiency can be significantly affected by solution’s temperature. These effects can be verified by studying the thermodynamic nature of the adsorbate-adsorbent system. The important thermodynamic parameters were ∆H°, ∆S° ∆G°, and E_a_. The Van’t Hoff relationship (Eq. 21) was used to calculate ΔH° (kJ/mol) and ΔS° (kJ/mol. K).

21 $$ln K_{c} = \frac{\Delta S^\circ }{R} - \frac{\Delta H^\circ }{{RT}}$$

where dimensionless equilibrium constant, universal gas constant (8.314 J/mol. K), and the RBBR solution temperatures are denoted as K_c_, R, and T, respectively. The following Eq. (22) was used to determine K_c_ value^55^:

22 $$K_{c} = \frac{{1000\frac{mg}{g} \times K_{L} \times\,\, molecular\,\, weight\,\, of \,\,adsorbate \times \left[ {adsorbate} \right]^{^\circ } }}{\gamma }$$

where standard concentration of adsorbate (can be assumed to be 1 mol/L at standard condition), dimensionless activity coefficient parameter, and Langmuir adsorption constant are denoted by [adsorbate]°, *ϒ* and K_L_ respectively. The other two parameters, ΔG° (kJ/mol) and E_a_ (kJ/mol), were computed using Eqs. (23 and 24), respectively:

23 $$\Delta G^\circ = \Delta H^\circ - T\Delta S^\circ$$

24 $$ln \,k_{2} = ln A - \frac{{E_{a} }}{RT}$$

where the PSO rate constant (g mg^−1^ min^−1^) and Arrhenius factor are denoted by k_2_ and A, respectively.

The collection of date palm stones complied with relevant institutional, national, and international guidelines and legislation.

## Conclusions

DPSAC was successfully synthesized from date palm stones using KOH/NaOH and microwave irradiation. Characterization of DPSAC showed a BET surface area of 715.30 m^2^/g, Langmuir surface area of 1061.93 m^2^/g, total pore volume of 0.39 cm^3^/g, and 2.15 nm. Based on the FTIR analysis, vinyl C–H out-of-plane bending, secondary alcohol, C–O stretching, phenol or tertiary alcohol, OH bending, and methyl C–H asymmetry were observed. Adsorption equilibrium showed that increasing initial concentration of RBBR from 25 to 300 mg/L led to increasing the adsorption of RBBR from 24.54 to 248.54 mg/g and decreasing the percentage removal from 98.16 to 82.85%. Adsorption of RBBR onto DPSAC was optimal at pH 3 with 71.85 mg/g and at solution temperature of 30 °C with 248.54 mg/g; the best isotherm and kinetic models for describing this adsorption process were the Freundlich and PFO models, respectively. Thermodynamic studies demonstrated that adsorption is naturally exothermic, spontaneous, and physisorption-controlled.

## Data Availability

The dataset generated in this study can be made available from the corresponding author upon a reasonable request.
